# Low-dose radiotherapy for greater trochanteric pain syndrome—a single-centre analysis

**DOI:** 10.1007/s00066-023-02107-4

**Published:** 2023-08-14

**Authors:** Michal Staruch, Silvia Gomez, Susanne Rogers, Istvan Takacs, Thomas Kern, Sabine Adler, Dieter Cadosch, Oliver Riesterer

**Affiliations:** 1 Center for Radiation Oncology KSA-KSB, Kantonsspital Aarau, 5001 Aarau, Switzerland; 2 Clinical Trial Unit, Department of Clinical Research, University Hospital of Basel, University of Basel, 4031 Basel, Switzerland; 3 Department of Radiation Oncology, Inselspital, Bern University Hospital, University of Bern, Bern, Switzerland; 4 Center for Radiation Oncology KSA-KSB, Kantonsspital Baden, 5404 Baden, Switzerland; 5 Department of Rheumatology, Kantonsspital Aarau, 5001 Aarau, Switzerland; 6 Department of Orthopaedic and Traumatology, Kantonsspital Aarau, 5001 Aarau, Switzerland

**Keywords:** Radiotherapy, Trochanteric bursitis, Greater trochanteric pain syndrome, Low-dose radiation therapy, Benign disease

## Abstract

**Purpose:**

To determine predictive factors associated with a good response (GR) to and efficacy of low-dose radiotherapy (LDRT) in patients with greater trochanteric pain syndrome (GTPS).

**Methods:**

Patients with GTPS were irradiated on a linear accelerator with 0.5–1.0 Gy per fraction to a total dose of 3.0–4.0 Gy per series. The endpoint was subjective good response (GR) to treatment 2 months after completion of the last LDRT series, defined as complete pain relief or marked improvement assessed using the von Pannewitz score. A positive response to steroid injection (SI) was defined as pain relief of at least 7 days. Patient and treatment-related characteristics were evaluated with respect to LDRT outcomes.

**Results:**

Outcomes were assessed for 71 peritrochanteric spaces (PTSs; 65 patients, 48 females, with mean age of 63 [44–91] years). Prior SI had been given to 55 (77%) PTSs and 40 PTSs received two series of LDRT. Two months after completion of LDRT, GR was reported in 42 PTSs (59%). Two series of LDRT provided a significantly higher rate of GR than one series (72.5 vs. 42% PTSs, *p* = 0.015). Temporary pain relief after prior SI predicted GR to LDRT compared with PTSs which had not responded to SI (73 vs. 28% PTSs, *p* = 0.001). A regional structural abnormality, present in 34 PTSs (48%), was associated with a reduction of GR to LDRT (44 vs. 73% PTSs, *p* = 0.017).

**Conclusion:**

LDRT is an effective treatment for GTPS. Administration of two LDRT series, prior response to SI, and absence of structural abnormalities may predict significantly better treatment outcomes.

## Introduction

Chronic pain and tenderness in the lateral aspect of the hip are relatively common clinical features, with a prevalence of up to 25% in the general population [1] and a preponderance in females (60%) [2], the latter likely due to female pelvic anatomy and tighter iliotibial bands [3, 4]. This clinical picture is commonly summarized as greater trochanteric pain syndrome (GTPS) and was formerly known as a trochanteric bursitis. For diagnosis of GTPS, physical examination remains the gold standard [4] and typically reveals a positive Trendelenburg test [5], reduced 30‑s single-leg stance, and resistant external de-rotation tests as well as a positive FABERE (flexion, abduction, external rotation, extension) test [6]. Most frequent radiological findings include trochanteric bursitis and gluteal tendinopathy, with an incidence ranging from 4 to 46% and from 18 to 50%, respectively [7]. Notably, the co-existence of the two aforementioned magnetic resonance imaging (MRI) findings [8] with other pathologies is not uncommon. In addition, due to the overlapping symptomatology of GTPS with, e.g., lumbar radiculopathy [9, 10], GTPS seems to be both underdiagnosed and misdiagnosed, potentially having social and economic consequences [11].

The aetiology of GTPS is still debated and a variety of conditions purportedly contribute to its pathogenesis. Although the exact pathogenetic mechanism remains to be elucidated, it is plausibly due to tears of gluteus medius and/or minimus tendons and friction between them, their bursae, the iliotibial fascia and the trochanter [12], all leading to disorganization of the collagen bundles. In addition, hypercellularity, increased proteoglycan synthesis and neovascularization are contributory [13, 14]. The most prevalent differential diagnoses encompass osteoarthritis of the hip joints, lumbar radiculopathy, rheumatoid arthritis, external coxa saltans or, less often and indirectly via altered biomechanics, discrepancy in leg length, pes planus, and genu varum or valgum [6].

Diverse treatment approaches are adopted for GTPS and include the use of anti-inflammatory analgesics and opiates, local injection of corticosteroids and local anaesthetics, physical therapy with infrared rays, shock waves, ultrasound, cryotherapy and thermotherapy [15, 16]. In patients who do not respond to the aforementioned conservative measures, low-dose radiotherapy (LDRT)—usually with six fractions (0.5–1.0 Gy per fraction)—is often the last conservative treatment modality to be tried for the persistent tendinitis. Its likely effectiveness has already been demonstrated in various conditions with active inflammation such as painful plantar fasciitis, achillodynia or painful elbow syndromes [17], albeit to a large part in retrospective analyses of large patient cohorts, thus establishing its place in clinical practice [11]. The predominant mechanisms by which LDRT exerts its biological effects include inhibition of mononuclear leucocyte adhesion, induction of apoptosis and the resultant blockade of various inflammatory pathways [18, 19].

As data regarding efficacy of LDRT in GTPS remain relatively scarce, we aimed to add to the evidence with our patient series. Defining the role of LDRT in modern management of GTPS may facilitate both decision-making before initiation of radiation treatment in patients suffering from refractory hip pain and provide a rationale for delaying surgical procedures [20].

## Methods

### Study design

This present study was a single-centre retrospective analysis which sought to identify predictive factors associated with a good response (GR) to LDRT in patients with persistent GTPS. The study was reviewed and approved by the regional ethics committee (northwest and central Switzerland, approval no.; 2020-02932).

### Patients and treatment

Patients irradiated between May 2015 and January 2021 twice a week on a linear accelerator with 0.5–1.0 Gy per fraction using opposing fields with 6‑MV photons to a total dose of 3.0–4.0 Gy per series were included. SIs were administered by general practitioners and usually consisted of triamcinolone acetate (40–80 mg) combined with lidocaine or bupivacaine. Patients received up to four steroid injections (SIs), with the last injection at least 6 months prior to LDRT. A positive response to SI was defined as pain alleviation of at least 7 days. CT-based treatment planning was used in all cases. The shared decision-making to perform a second series was individualized and depended on the pain response 2 months after completion of the initial series.

### Study endpoint

Pain response to LDRT was evaluated according to the von Pannewitz score (VPS), which classifies responses into four categories: complete pain relief, marked improvement, slightly improved, unchanged. The definition of GR defined by complete pain relief or marked improvement was adopted as described previously by others [21, 22]. GR was routinely assessed 2 months after completion of the last LDRT series in all patients and was used as the study endpoint.

### Data collection and statistical analysis

The data on treatment and patient characteristics were retrospectively collected in an electronic database. SPSS statistical package 20.0 (IBM Corp., Armonk, NY, USA) was used for the statistical evaluation. Descriptive statistics were calculated for continuous and categorical variables, and were presented as mean for continuous variables and frequencies for categorical variables. Fisher’s exact test for categorical variables was used to test for between-group differences. All *p*-values were derived from two-sided statistical tests, and *p* < 0.05 was considered statistical significance. Multivariate logistic regression models were created to determine the predictors of LDRT outcome. Odds ratios and confidence intervals were calculated to evaluate the potential predictors.

## Results

### Patient and treatment characteristics

**Fig. 1:**
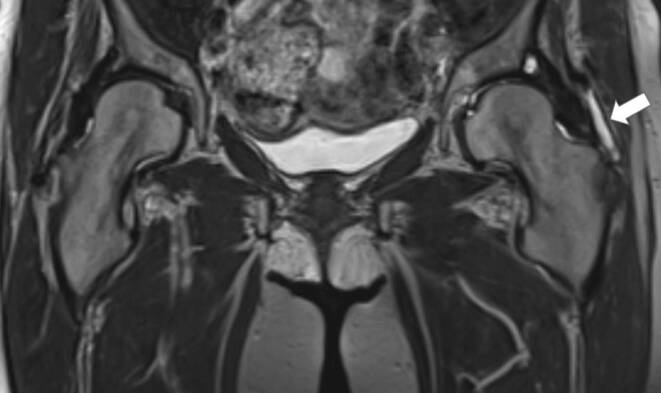
Left subgluteal bursitis (*white* *arrow*) diagnosed with MRI

Overall, 65 consecutive patients with a median age of 63 years (44–91 years) diagnosed with GTPS and refractory to conventional therapy were included. The patients, of whom 48 (74%) were female and 17 (26%) were male, underwent treatment with LDRT within the study period. A concomitant chronic rheumatological condition was reported in 20% (13/65) of the patients. Prior SI had been performed in 55 peritrochanteric spaces (PTSs; 77%) with a pain alleviation of at least 7 days in 67% of injected PTSs. In 42 PTSs (58%), diagnostic workup was supplemented by magnetic resonance imaging (MRI; Fig. 1). All MRI examinations included axial and coronal T1 and T2 as well as proton density fat-suppression sequences. Contrast enhancement was administered only in a limited number of cases. The scanned area encompassed standard hip and pelvic regions. When a radicular component was suspected, MRI with a lumbar spine scan was also conducted. Trochanteric/subgluteal bursitis was radiologically confirmed in 33 (47%) GTPS cases. Notably, the presence of gluteal tendinopathy, other regional structural abnormalities (i.e., coxarthrosis, sacroiliac joint dysfunction and/or femoroacetabular impingement) and lumbar radiculopathy was described in 56 (79%), 34 (48%) and 41 PTSs (58%), respectively (Table 1).

**Table 1:** Patient and treatment characteristics

Variables	
*Patients*	*Total number of patients: 65*
Gender (M:F)	17:48
Age (years), median (range)	6 (44–91)
Rheumatic disease (%)	13, (20)
*Treated PTSs*	*Total number of PTSs 71 (%)*
Prior treatment with SI	55 (77)
Temporary pain alleviation after SI (≥ 7 days)	37 (52)
Previous diagnosis of coxarthrosis, sacroiliac joint dysfunction and/or femoroacetabular impingement	34 (48)
Total hip arthroplasty	15 (21.1)
Lumbar radiculopathy	41 (57.7)
MRI	42 (59.2)
Trochanteric bursitis (% of 42)	33 (78.6)
Regional tendinopathy (% of 42)	39 (92.9)
*Treatment characteristics*	*Total number of PTSs 71 (%)*
1 LDRT series	31 (44)
2 LDRT series	40 (56)
Dose per fraction 0.5 Gy	69 (97)
Dose per fraction 1.0 Gy	2 (3)
Total dose per series 3.0 Gy	68 (96)
Total dose per series 4.0 Gy	3 (4)
Total dose per treatment of 3.0 Gy	31 (44)
Total dose per treatment of 6.0 Gy	37 (52)
Total dose per treatment of 7.0 Gy	1 (1)
Total dose per treatment of 8.0 Gy	2 (3)
Median total dose (Gy)	6.0
Transient pain exacerbation during LDRT	14 (19.7)

*M* male, *F* female, *PTSs* peritrochanteric spaces, *SI* steroid injection, *MRI* magnetic resonance imaging, *LDRT* low-dose radiotherapy

Irradiation was applied to 71 PTSs in the 65 patients, unilaterally in 59/65 (91%) and bilaterally in 6/65 (9%) of patients. LDRT was performed with 0.5 Gy per fraction and 3.0 Gy per series, with the exception of two PTSs of two different patients that were treated with 1.0 Gy per fraction up to a total dose of 4.0 Gy per series. Two LDRT series were required in 40 PTSs (56%) with refractory GTPS.

### Treatment outcome

GR was achieved 2 months after completion of the initial series in 41 (58%) GTPS PTSs. The majority of the GTPS cases were treated with two LDRT series. GR was observed in 42% (13 of 31 PTSs) after one series versus in 73% (29 of 40 PTSs) of patients who received two series. The study endpoint was met in 59% of the treated PTSs. In 10% of cases with GR reported after the initial series (4 of 41 PTSs), the treatment benefit was only temporary and waned after 2 months of follow-up and could not be achieved with a second series. Notably, in the subgroup with gluteal tendinopathy, we observed a GR rate of 55% (Table 2).

**Table 2:** Good response rate in treated PTSs

Variables	Number of PTSs (total 71; %)	Good response rate (%)	*p-*value
1 LDRT series	31 (44)	13 (42)	0.015*
2 LDRT series	40 (56)	29 (72.5)	
Prior treatment with SI	55 (77)	33 (60)	0.5
Temporary pain relief after SI (≥ 7 days)	37 (52)	27 (73)	0.009*
Presence of coxarthrosis, sacroiliac joint dysfunction and/or femoroacetabular impingement	34 (48)	15 (44.1)	0.017*
Total hip arthroplasty	15 (21.1)	6 (40)	0.138
Lumbar radiculopathy	41 (57.7)	23 (56.1)	0.628
*PTSs with MRI images*	42 (59.2)	24 (57.1)	0.807
Trochanteric bursitis (% of 42)	33 (78.6)	21 (63.6)	0.629
Regional tendinopathy (% of 42)	39 (92.9)	23 (59)	0.567

The difference between subgroups was investigated using Fisher’s exact test

*Significance level *p* < 0.05

*PTSs* peritrochanteric spaces, *SI* steroid injection, *MRI* magnetic resonance imaging, *LDRT* low-dose radiotherapy

### Logistic regression analysis

**Fig. 2:**
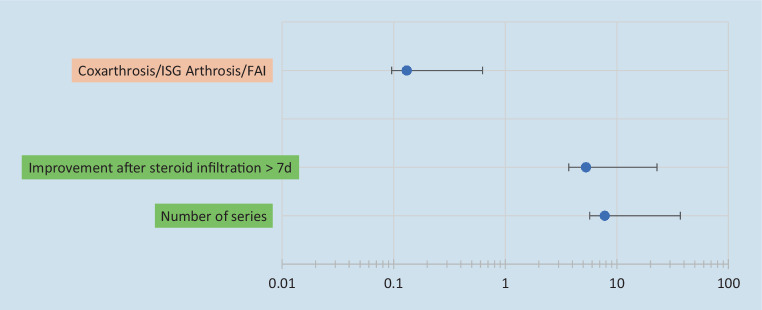
Results of logistic regression analysis (odds ratios) of prognostic factors of good response. *FAI* femoroacetabular impingement, *ISG* sacroiliac joint

The variables with a statistically significant association (*p*-value < 0.05) were included in logistic regression. Completing two series of LDRT (*p* = 0.015) and a temporary response to SI (*p* = 0.009) were associated with higher GR rate. These variables were also confirmed to be positive predictors of treatment success, with an odds ratio (OR) of 7.8 (confidence interval 95% [CI]: 2.1–29.3) for two series of LDRT and OR = 05.3 (CI: 1.6–17.6) for previous steroid response, respectively. Conversely, a concomitant regional structural abnormality had a significant negative predictive value, with OR = 0.1 (CI: 0.04–0.49, *p* = 0.017; 3, Fig. 2).

**Table 3:** Results of logistic regression analysis

Variables	*p*-value	OR (95% CI)
Number of series: 2 vs. 1 series	< 0.05	7.8 (2.1–29.3)
Temporary pain relief after steroid injection (≥ 7 days)	< 0.05	5.3 (1.6–17.6)
Presence of coxarthrosis, sacroiliac joint dysfunction and/or femoroacetabular impingement	< 0.05	0.131 (0.035–0.495)

Only variables with a statistically significant association with a good response are shown

## Discussion

The efficacy of low-dose radiotherapy has been demonstrated in large retrospective analyses across a wide spectrum of non-malignant joint disorders. LDRT provides an excellent safety profile with negligible adverse effects such as transient pain exacerbation, and thus offers a cost-effective alternative to orthopaedic surgical interventions [17, 22]. Similar to previous reports, in our cohort, the efficacy endpoint (GR rate) could be achieved in a clear majority of refractory GTPS cases (59%), whereas a transient pain flare was observed in 14 cases (20%) [11].

The present study sought to define whether baseline clinical characteristics can be useful for decision-making in refractory GTPS. Surgery is the treatment modality commonly recommended in the management of persistent GPTS. Undoubtedly, if used with well-defined indications and especially in low-risk patients, surgery can help the patients [15, 16]. Nevertheless, particularly in elderly patients with relevant comorbidities and an increased surgical risk, surgery should be the last resort [21, 23]. Importantly, the elderly and comorbid populations are growing substantially in developed countries, and therefore our cohort is representative of those who may benefit from LDRT [21, 24].

For successful treatment of GTPS with LDRT, patient selection is very important. As described by Wilson et al. [8], MRI is the imaging modality of choice in the diagnostic workup of GTPS patients. MRI serves not only to exclude spine- and/or hip-related pathology mimicking GTPS, but also to specify the exact underlying condition. Noteworthily, in our study, the majority of GTPS cases (42 PTSs) were investigated with MRI. Interestingly, it was demonstrated that isolated trochanteric bursitis without concomitant pathologies such as gluteal tendinopathy or tears is positively correlated with effective pain alleviation following SI [8]. This report and other studies [22, 25] support our findings that co-existence of regional structural abnormalities diminishes the benefits associated with LDRT. In addition, routine supplementation of the clinical diagnosis with imaging seems to be the recommended pathway as radiological findings, which prognosticate lower response rate, should be carefully considered before proceeding with LDRT [5, 8]. A thorough evidence-based workup may therefore help to avoid unnecessary delays in definitive surgical management.

The finding that response to prior SI strongly predicts LDRT response supports the contribution of an active inflammatory component as part of the trochanteric region pain syndromes [8, 26]. Both LDRT and SIs act primarily as anti-inflammatory agents, although these two therapeutic modalities enact this role differently at cellular and molecular levels [26]. LDRT affects, among others, endothelial cells, thus decreasing adhesion of leukocytes and monocytes as well as inducing production of anti-inflammatory cytokines (e.g., interleukin 10) [27]. Of particular interest, in our cohort, the application of two LDRT series with a 2-month interval seems to offer a significantly greater chance of achieving GR in comparison to one series only. The simple explanation would be accumulation of anti-inflammatory effects from both series. However, when considering the pathogenetic mechanism of repetitive microtrauma underlying GTPS, transient pain exacerbation following treatment and necessary modification of concomitant analgesia add to the complexity of the clinical scenario under which the decision to proceed with a second LDRT series is to be made. Acknowledging that follow-up was limited to 2 months after the last LDRT series, that the cohort size was relatively small and the fact of the somewhat contradictory previous reports on the subject [17, 21, 22, 28, 29], we advise individualisation of the therapeutic strategy with regard to the number of sessions needed in each case, until more robust data can be gathered.

Our study has several limitations, mainly due to its retrospective nature and the limited number of studied subjects. In addition, we could not exclude the impact of a placebo effect and modifications of concomitant analgesia (predominantly due to the lack of prospective documentation and analgesic requirement for other indications) on the definitive effect of LDRT. However, these factors are also probably underestimated in studies on efficacy of other treatment modalities of GTPS. Changes in quality of life and a possible gain in function as a result of irradiation could not be analysed retrospectively. Furthermore, pain relief was not consistently documented using the numerical rating scale or visual analogue scale, which could further objectify the results. Finally, we could only evaluate the short-term response to LDRT. Long-term follow-up will provide valuable data as to the durability of the results of LDRT, nonetheless at the price of accumulating confounding factors.

## Conclusion

The findings of the present study provide additional evidence that LDRT is a reasonably effective treatment modality in GTPS. Our data offer a further rationale for conducting a randomised study investigating the use of LDRT for treatment of GTPS.
