# Correction to: Low-dose radiotherapy for greater trochanteric pain syndrome—a single-centre analysis

**DOI:** 10.1007/s00066-024-02273-z

**Published:** 2024-08-02

**Authors:** Michal Staruch, Silvia Gomez, Susanne Rogers, Istvan Takacs, Thomas Kern, Sabine Adler, Dieter Cadosch, Oliver Riesterer

**Affiliations:** 1 Center for Radiation Oncology KSA-KSB, Kantonsspital Aarau, 5001 Aarau, Switzerland; 2 Clinical Trial Unit, Department of Clinical Research, University Hospital of Basel, University of Basel, 4031 Basel, Switzerland; 3 Department of Radiation Oncology, Inselspital, Bern University Hospital, University of Bern, Bern, Switzerland; 4 Center for Radiation Oncology KSA-KSB, Kantonsspital Baden, 5404 Baden, Switzerland; 5 Department of Rheumatology, Kantonsspital Aarau, 5001 Aarau, Switzerland; 6 Department of Orthopaedic and Traumatology, Kantonsspital Aarau, 5001 Aarau, Switzerland

**Correction to:**

**Strahlenther Onkol 2023**

10.1007/s00066-023-02107-4

In this article the affiliation details of affiliation 3 were incorrectly given as ‘University of Bern, Hochschulstrasse 6, 3012 Bern, Switzerland’ but should have been ‘Department of Radiation Oncology, Inselspital, Bern University Hospital, University of Bern, Bern, Switzerland’.

The original article has been corrected.

